# Simplified large African carnivore density estimators from track indices

**DOI:** 10.7717/peerj.2662

**Published:** 2016-12-22

**Authors:** Christiaan W. Winterbach, Sam M. Ferreira, Paul J. Funston, Michael J. Somers

**Affiliations:** 1 Centre for Wildlife Management, University of Pretoria, Pretoria, South Africa; 2 Tau Consultants (Pty) Ltd, Maun, Botswana; 3 Scientific Services, SANParks, Skukuza, South Africa; 4 Panthera, Kongola, Namibia; 5 Centre for Invasion Biology, University of Pretoria, Pretoria, South Africa

**Keywords:** Survey technique, Lion, Leopard, Cheetah, Spotted hyena, Brown hyena, Wild dog, Conservation, Spoor, AIC

## Abstract

**Background:**

The range, population size and trend of large carnivores are important parameters to assess their status globally and to plan conservation strategies. One can use linear models to assess population size and trends of large carnivores from track-based surveys on suitable substrates. The conventional approach of a linear model with intercept may not intercept at zero, but may fit the data better than linear model through the origin. We assess whether a linear regression through the origin is more appropriate than a linear regression with intercept to model large African carnivore densities and track indices.

**Methods:**

We did simple linear regression with intercept analysis and simple linear regression through the origin and used the confidence interval for ß in the linear model *y* = *αx* + ß, Standard Error of Estimate, Mean Squares Residual and Akaike Information Criteria to evaluate the models.

**Results:**

The Lion on Clay and Low Density on Sand models with intercept were not significant (*P* > 0.05). The other four models with intercept and the six models thorough origin were all significant (*P* < 0.05). The models using linear regression with intercept all included zero in the confidence interval for ß and the null hypothesis that ß = 0 could not be rejected. All models showed that the linear model through the origin provided a better fit than the linear model with intercept, as indicated by the Standard Error of Estimate and Mean Square Residuals. Akaike Information Criteria showed that linear models through the origin were better and that none of the linear models with intercept had substantial support.

**Discussion:**

Our results showed that linear regression through the origin is justified over the more typical linear regression with intercept for all models we tested. A general model can be used to estimate large carnivore densities from track densities across species and study areas. The formula *observed track density = 3.26 × carnivore density* can be used to estimate densities of large African carnivores using track counts on sandy substrates in areas where carnivore densities are 0.27 carnivores/100 km^2^ or higher. To improve the current models, we need independent data to validate the models and data to test for non-linear relationship between track indices and true density at low densities.

## Introduction

Africa has seven large carnivores: lion *Panthera leo* (Linnaeus, 1758), leopard *Panthera pardus* (Linnaeus, 1758), spotted hyaena *Crocuta crocuta* (Erxleben, 1777), brown hyaena *Parahyaena brunnea* (Thunberg, 1820), striped hyaena *Hyaena hyaena* (Linnaeus, 1758), cheetah *Acinonyx jubatus* (Schreber, 1775) and wild dog *Lycaon pictus* (Temminck, 1820). The range, population size and trend of these large carnivores are important parameters to assess their status globally (Bauer et al., 2015; Wiesel, 2015). These parameters are used to plan conservation strategies at different scales ranging from the entire distribution range of hyaena (Mills & Hofer, 1998), cheetah and wild dog (Durant, 2007), to regional conservation plans for lion (IUCN/SSC, 2006) and national plans for cheetah and wild dog (Lindsey & Davies-Mostert, 2009).

Methods used to estimate densities of African large carnivores include intensive studies (Smuts, 1982; Maude, 2010), call in surveys (Cozzi et al., 2013; Mills, Juritz & Zucchini, 2001; Ogutu & Dublin, 1998), camera trap surveys (Balme, Hunter & Slotow, 2009; Kent & Hill, 2013), track counts (Funston et al., 2010; Keeping & Pelletier, 2014; Stander, 1998) and measuring track dimensions to identify individuals (Gusset & Burgener, 2005). This paper focuses on the use of track indices to estimate large carnivore densities in Africa.

A previous study (Stander, 1998) demonstrated a significant linear correlation between true density and track density for leopard, lion and wild dog; which can be used to estimate carnivore densities (animals/100 km^2^) from track densities (tracks/100 km). The leopard model is based on a bootstrap analysis to simulate different leopard densities using known individuals from a known population at one study area (Stander, 1998). The lion and wild dog model used densities from two sites (four data points). The slope of the regression for leopard was different to that of lion and wild dog (Stander, 1998), showing potential differences in the track density–true density relation for species and sites.

Funston et al. (2001) provided a calibration for lion in the Kgalagadi Transfrontier Park (southern Botswana and South Africa) and showed the potential to estimate large carnivore densities from track counts, using one general model. Houser, Somers & Boast (2009) did track density–true density calibration for cheetah in southern Botswana, but unfortunately their estimate of true density was flawed and thus their calibration is invalid. Funston et al. (2010) did the first analysis for multiple species and localities and provided models to assess population size and trends of large carnivores from track-based surveys on sandy and clay soils. This calibration included data for five of the seven large carnivores in Africa and spanned 18 different study sites from seven study areas in Namibia, Botswana, South Africa, Zimbabwe, Kenia and Tanzania (Funston et al., 2010). Some recent studies used these models to assess large carnivore densities in parts of Botswana (Bauer et al., 2014; Boast & Houser, 2012; Ferreira, Govender & Herbst, 2013; Kent & Hill, 2013). Refer to Funston et al. (2010) for the protocols to conduct track count surveys.

The formula to estimate large carnivore densities using the general model on sandy soils is *y* = 3.15*x* + 0.4, where *y* is track density (tracks/100 km) and *x* is carnivore density (animals/100 km^2^) (Funston et al., 2010). This formula would yield negative density estimates below track densities of 0.4 tracks/100 km, for example carnivore density would be estimated as −0.06 animals per 100 km^2^ from a track density of 0.2 tracks per 100 km. Boast & Houser (2012) resolved this problem for leopard by using the formula from Stander (1998) to estimate leopard densities at low track densities. Williams (2011) and Williams et al. (2016) opted to use the lion and wild dog model from Stander (1998) to estimate carnivore densities, although this model is based on only four data points.

Whereas Stander (1998) used linear models through the origin, Funston et al. (2010) followed the more conventional approach of a linear model with intercept (Eisenhauer, 2003; Quinn & Keough, 2002; Sokal & Rohlf, 1995). Although biology may dictate that there should be no tracks if no carnivores are present (i.e., we expect *Y* = 0 when *X* = 0), the regression may not intercept at zero. Imperfect detection of tracks (Mackenzie, 2006) at low densities may result in such a biological anomaly, or the relationship may not be linear with values approaching zero (Quinn & Keough, 2002). In such a case, Quinn & Keough (2002) recommended using a model with intercept that fits the data better, even if it does not intercept at zero. Although there are circumstances where regression through the origin is appropriate (Quinn & Keough, 2002; Sokal & Rohlf, 1995), Eisenhauer (2003) described the use of regression through the origin as “a subject of pedagogical neglect, controversy and confusion”.

We used the guidelines from Sokal & Rohlf (1995), Quinn & Keough (2002) and Eisenhauer (2003) to assess whether a linear model fitted through the origin is more appropriate for the dataset from Funston et al. (2010) than the linear model with intercept that they used. We demonstrate the impact of using different models to estimate population size at various track densities.

## Methods

We repeated the simple linear regression with intercept analysis done by Funston et al. (2010) and extended it to include simple linear regression through the origin. The data used by Funston et al. (2010) is summarized in Table 1. For their analysis of carnivores on sandy soils Funston et al. (2010) used the lion data (record 1–10 in Table 1) (Model 1) and then Model 2 “a combined model for all carnivore species on sandy soils” (record 1–16). Although they called it “all carnivore species” they excluded the data points they had for brown hyaena (Table 2). Also included in Table 2 are the data from Stander (1998) for leopard density, and track density for the site he labeled “Experimental”. Funston et al. (2010) included this leopard density without the track density in their table of mean densities for the respective large carnivores. We did an additional model for all the carnivores on sandy soils that included the data from Table 1 (record 1–16) and Table 2 (record 25–27). For clarity we will refer to this as Model 3 General Carnivores on Sand. Models 4 and 5 are Lion on Clay and Lion plus Cheetah on Clay. Model 6 Low Density on Sand is a subset of carnivore densities below 1 animal/100 km^2^ for sandy substrates.

**Table 1:** Mean density and tracks density of respective carnivores used in linear regression models by Funston et al. (2010).

Record number	Substrate	Location	Species	Density individuals/100 km^2^	Density tracks/100 km
1	Sandy	Dune-north	Lion	0.67	1.6
2	Sandy	Dune-south	Lion	0.95	2.9
3	Sandy	Sesatswe	Lion	1.35	5.5
4	Sandy	Mabuasehube	Lion	1.68	4.5
5	Sandy	Mosimane	Lion	2.2	7.2
6	Sandy	Main camp	Lion	2.73	9.5
7	Sandy	Venetia	Lion	3.3	9.7
8	Sandy	El Karama	Lion	5.8	18.2
9	Sandy	Mugie	Lion	6	17.8
10	Sandy	Mpala	Lion	6.15	22.5
11	Sandy	Dune-north	Cheetah	0.54	1.7
12	Sandy	Dune-south	Cheetah	0.54	4.9
13	Sandy	Dune-north	Leopard	0.27	0.8
14	Sandy	Dune-south	Leopard	0.27	0.4
15	Sandy	Dune-north	Spotted Hyaena	0.9	4.7
16	Sandy	Dune-south	Spotted Hyaena	0.9	3.4
17	Clay	Short-grass dry	Lion	7	1.5
18	Clay	Short-grass wet	Lion	20	10.5
19	Clay	Long-grass wet	Lion	21.08	8
20	Clay	Long-grass dry	Lion	24.28	16.5
21	Clay	Short-grass dry	Cheetah	2.26	1.0
22	Clay	Long-grass wet	Cheetah	2.29	0.9
23	Clay	Short-grass wet	Cheetah	6.78	9.0
24	Clay	Long-grass dry	Cheetah	9.16	1.6

**Table 2:** Additional mean density and tracks density of brown hyaena from Funston et al. (2010) and leopard from Stander (1998).

Record number	Substrate	Location	Species	Density individuals/100 km^2^	Density tracks/100 km
25	Sandy	Dune-south	Brown Hyaena	1.6	5.2
26	Sandy	Dune-north	Brown Hyaena	1.6	6.4
27	Sandy	Experimental	Leopard	1.45	2.62

Forcing the model through the origin is rarely appropriate (Quinn & Keough, 2002, page 110), therefore we used the criteria in Table 3 to assess if linear regression through origin is justified over linear regression with intercept (Eisenhauer, 2003; Quinn & Keough, 2002; Sokal & Rohlf, 1995). There is some justification to fit a linear model through the origin if *Y* = 0 when *X* = 0, and the null hypothesis that ß =0 is not rejected (Quinn & Keough, 2002, page 99). This warrants further investigation using Standard Error of Estimate and Mean Square Residual (Table 3). We also calculated corrected Akaike Information Criteria (AIC_c_) to assist model selection between intercept and through the origin models: AIC_c_ = *n*ln(SS residual∕*n*) + 2*K* + (2*K*(2*K* + 1))∕(*n* − *K* − 1), where *n* is sample size and *K* is the total parameters in the model including intercept and *σ*^2^ (Burnham & Anderson, 2004). A non-linear relationship with values approaching zero is possible (Quinn & Keough, 2002). We added a logarithmic curve fitted as part of Model 6 to test for a non-linear relationship at low densities.

**Table 3:** Criteria to assess the use of linear regression through origin over linear regression with intercept.

Criteria	Source
*Y* = 0 when *X* = 0	(Eisenhauer, 2003; Quinn & Keough, 2002, page 99)
Null hypothesis that ß = 0 is not rejected based on *P* value for ß; Confidence interval for ß in the linear model *y* = *αx* + ß includes zero	(Eisenhauer, 2003; Quinn & Keough, 2002, page 99)/(Sokal & Rohlf, 1995, page 474)
Mean Squares Residual is smaller for regression through the origin than regression with intercept, indicating a better fit.	(Quinn & Keough, 2002, page 99)
Standard error is smaller for regression through the origin than regression with intercept, indicating a better fit.	(Eisenhauer, 2003)

We compared population estimates derived from the leopard model (Stander, 1998), Model 2 Carnivores on Sand (regression with intercept) (Funston et al., 2010) and Model 3 General Carnivores on Sand (regression through origin). We used track densities from recently published studies to estimate carnivore density before calculating population estimates for a hypothetical study area of 10,000 km^2^. The difference between population estimates using Model 3 General Carnivores on Sand model (regression through origin) and Model 2 Carnivores on Sand (regression with intercept) were calculated as a percentage of population estimate from Model 3 General Carnivores on Sand model (regression through origin).

## Results

Regressions through the origin were significant (*P* < 0.05) for the six models tested (Table 4). Regression analyses with intercept were statistically significant at *P* < 0.05 except for Model 4 Lion on Clay with intercept and Model 6 Low Density on Sand with intercept (Table 4) that were not significant. Model 6 Low Density on Sand logarithmic was not significant (*t*_*i*_ = 3.86 + (2.32*x*ln(*x*_*i*_)), *F*_1,6_ = 5.587, *P* = 0.056, *R*^2^ = 0.482). Eight data points, two each for lion, leopard, spotted hyaena and cheetah were used in Model 6 Low Density on Sand.

**Table 4:** Summary of linear regression with intercept and through the origin for carnivore density (predictor) and track density (dependent) on sandy and clay soils. R square^*a*^ measures the proportion of variation in the data described by the linear regression with intercept. R square^*b*^ measures the proportion of the variability in the dependent variable about the origin explained by regression through the origin. This cannot be compared to R square^*a*^.

Model	Description	Linear regression	*F* value	Significance	R square^*a*^	R square^*b*^
Model 1	Lion sandy soil with intercept	*t*_*i*_ = 3.3*x*_*i*_ − 0.31	*F*_1,8_ = 244.914	*P* < 0.001	0.972	
	Lion sandy soil through origin	*t*_*i*_ = 3.23*x*_*i*_	*F*_1,9_ = 819.856	*P* < 0.001		0.990
Model 2	Carnivores sandy soil with intercept	*t*_*i*_ = 3.16*x*_*i*_ + 0.42	*F*_1,14_ = 333.281	*P* < 0.001	0.962	
	Carnivores sandy soil through origin	*t*_*i*_ = 3.26*x*_*i*_	*F*_1,15_ = 732.137	*P* < 0.001		0.981
Model 3	General carnivores on sand with intercept	*t*_*i*_ = 3.18*x*_*i*_ + 0.31	*F*_1,18_ = 356.600	*P* < 0.001	0.954	
	General carnivores on sand through origin	*t*_*i*_ = 3.26*x*_*i*_	*F*_1,19_ = 850.826	*P* < 0.001		0.979
Model 4	Lion on clay with intercept	*t*_*i*_ = 0.75*x*_*i*_ − 4.34	*F*_1,3_ = 9.998	*P* > 0.05	0.833	
	Lion on clay through origin	*t*_*i*_ = 0.53*x*_*i*_	*F*_1,4_ = 44.847	*P* < 0.01		0.937
Model 5	Lion and Cheetah on clay with intercept	*t*_*i*_ = 0.55*x*_*i*_ − 0.28	*F*_1,7_ = 14.695	*P* < 0.01	0.710	
	Lion and Cheetah on clay through origin	*t*_*i*_ = 0.54*x*_*i*_	*F*_1,8_ = 47.940	*P* < 0.001		0.873
Model 6	Low density on sandy soil with intercept	*t*_*i*_ = 4.10*x*_*i*_ − 0.03	*F*_1,6_ = 4.615	*P* > 0.05	0.435	
	Low density on sandy soil through origin	*t*_*i*_ = 4.06*x*_*i*_	*F*_1,7_ = 37.116	*P* < 0.001		0.841

We used the criteria from Table 3 to assess and select between the models with linear regression with intercept and regression through the origin. Zero tracks are expected when zero carnivores are present, complying with the condition that *Y* = 0 when *X* = 0. The six models using linear regression with intercept all included zero in the confidence interval for ß  (Table 5). The *P* values for ß were not significant (*P* < 0.05), thus the null hypothesis that ß = 0 could not be rejected for all linear models with intercept (Table 5). The Mean Square Residual and Standard Error of Estimate (Table 6) for the linear model through the origin were smaller than the comparative linear model with intercept, indicating that the linear model through the origin provided a better fit than the linear model with intercept for all the models.

**Table 5:** Coefficients for linear regressions with intercept and linear regression through origin using density (predictor) and tracks (dependent). Standard error for coefficient, coefficient of variance, *t* value and level of significance are shown for each model coefficient.

Model	Description	Coefficient	Value	SE of coefficient	CV (%)	*t*	Significance level	Lower bound	Upper bound
Model 1	Lion sandy soil with intercept	Constant (b)	−0.31	0.8	258.1	−0.385	*P* = 0.711	−2.196	1.58
	Lion sandy soil with intercept	Rate of change (a)	3.3	0.21	6.4	15.65	*P* < 0.001	2.8	3.796
	Lion sandy soil through origin	Rate of change (a)	3.23	0.11	3.4	28.633	*P* < 0.001	2.971	3.491
Model 2	Carnivores sandy soil with intercept	Constant (b)	0.42	0.51	121.4	0.813	*P* = 0.431	−0.69	1.523
	Carnivores sandy soil with intercept	Rate of change (a)	3.16	0.17	5.4	18.256	*P* < 0.001	2.785	3.532
	Carnivores sandy soil through origin	Rate of change (a)	3.26	0.12	3.7	27.058	*P* < 0.001	3	3.516
Model 3	General Carnivores on sand with intercept	Constant (b)	0.31	0.47	151.6	0.656	*P* = 0.521	−0.68	1.29
	General Carnivores on sand with intercept	Rate of change (a)	3.18	0.17	5.3	18.884	*P* < 0.001	2.83	3.54
	General Carnivores on sand through origin	Rate of change (a)	3.26	0.11	3.4	29.169	*P* < 0.001	3.03	3.5
Model 4	Lion on clay with intercept	Constant (b)	−4.34	4.53	104.4	−0.958	*P* = 0.439	−23.85	15.16
	Lion on clay with intercept	Rate of change (a)	0.75	0.24	32.0	3.162	*P* = 0.087	−0.27	1.76
	Lion on clay through origin	Rate of change (a)	0.53	0.08	15.1	6.697	*P* < 0.01	0.28	0.79
Model 5	Lion and Cheetah on clay with intercept	Constant (b)	−0.28	2.05	732.1	−0.137	*P* = 0.896	−5.299	4.737
	Lion and Cheetah on clay with intercept	Rate of change (a)	0.55	0.14	25.5	3.833	*P* < 0.01	0.2	0.904
	Lion and Cheetah on clay through origin	Rate of change (a)	0.54	0.08	14.8	6.924	*P* < 0.001	0.353	0.719
Model 6	Low density on sandy soil with intercept	Constant (b)	−0.03	1.30	4333.3	−0.025	*P* = 0.981	−3.208	3.143
	Low density on sandy soil with intercept	Rate of change (a)	4.10	1.91	46.6	2.148	*P* = 0.075	-.570	8.769
	Low density on sandy soil through origin	Rate of change (a)	4.06	0.69	17.0	6.092	*P* < 0.001	2.481	5.629

**Table 6:** Evaluation of linear regression models for carnivore density (predictor) and track density (dependent) on sandy and clay soils. Smaller values of standard error of estimate, mean square residual and small sample corrected Akaike Information Criteria (AIC_c_) indicate better fit of model.

Model	Description	Linear regression	Standard error of estimate	Mean square residual	AIC_c_	Δ_*i*_ Values	*ω*_*i*_
Model 1	Lion sandy soil through origin	*t*_*i*_ = 3.23*x*_*i*_	1.283	1.645	8.46	0.00	0.88
	Lion sandy soil with intercept	*t*_*i*_ = 3.3*x*_*i*_ − 0.31	1.357	1.841	12.54	4.08	0.12
Model 2	Carnivores sandy soil through origin	*t*_*i*_ = 3.26*x*_*i*_	1.381	1.907	13.12	0.00	0.76
	Carnivores sandy soil with intercept	*t*_*i*_ = 3.16*x*_*i*_ + 0.42	1.398	1.955	15.40	2.28	0.24
Model 3	General Carnivores on sand through origin	*t*_*i*_ = 3.26*x*_*i*_	1.352	1.828	15.18	0.00	0.77
	General Carnivores on sand with intercept	*t*_*i*_ = 3.18*x*_*i*_ + 0.31	1.374	1.888	17.56	2.38	0.23
Model 4	Lion on clay through origin	*t*_*i*_ = 0.53*x*_*i*_	3.063	9.379	23.80	0.00	–
	Lion on clay with intercept	*t*_*i*_ = 0.75*x*_*i*_ − 4.34	3.105	9.642	–	–	–
Model 5	Lion and Cheetah on clay through origin	*t*_*i*_ = 0.54*x*_*i*_	3.118	9.722	23.53	0.00	0.94
	Lion and Cheetah on clay with intercept	*t*_*i*_ = 0.55*x*_*i*_ − 0.28	3.363	11.307	29.10	5.58	0.06
Model 6	Low density on sandy soil through origin	*t*_*i*_ = 4.06*x*_*i*_	1.28	1.639	9.29	0.00	0.94
	Low density on sandy soil with intercept	*t*_*i*_ = 4.10*x*_*i*_ − 0.03	1.383	1.912	14.89	5.60	0.06

The value of *K* was three to calculate AIC_c_for models with intercept (one parameter plus intercept plus *σ*^2^) and two for models through the origin (one parameter plus *σ*^2^). Sample size for Model 4 Lion on Clay with intercept resulted in *K* = 0 and AIC_c_ could not be calculated. AIC_c_ values are used to compare models based on the same data set (Burnham & Anderson, 2004), for example different versions of Model 1, but cannot be used to compare among models 1–6. Models through the origin had the lowest AIC_c_ values and the Δ_*i*_ were between 2.28 and 5.60 (Table 6) for the models with intercept, indicating that none of the intercept models have substantial support (Δ_*i*_ > 2) (Burnham & Anderson, 2004).

We tested Model 3 General Carnivore on Sand through origin with data from a study area of 629 km^2^. At the time of the track survey there were 18 lions present at a density of 2.86 lions/100 km^2^. We surveyed 294 km and recorded 13 incidences of lions consisting of 22 individuals. Track frequency was 10 ± 1.5 km/lion (CV = 14.8%). Track density was 7.2 (95% CI [3.3–1.1.2]), resulting in a density estimate of 2.2 (1.0–3.4) lions/100 km^2^. This 78% of the true density and the 95% CI included the true density.

Table 7 provides a comparison of population estimates derived from three models. The leopard density estimates from Model 3 General Carnivore on Sand model is 58% of estimates calculated with the leopard model from Stander (1998). The difference in population estimates between Model 3 General Carnivore on Sand through origin and Model 2 Carnivore on Sand with intercept (Funston et al., 2010) is the largest at low densities (Table 7). At the lower limit for density extrapolation (0.88 tracks/100 km) the difference is 43.5% and converged to less than 10% difference at track density of 3.1 tracks/100 km. Using Model 6 Low Density on Sand through origin provided more conservative estimates that are 80% of estimates using Model 3 General Carnivore on Sand through origin.

**Table 7:** Comparison of carnivore population estimates for a reference area of 10,000 km, using different models to estimate density (animals/100 km^2^) from track densities (tracks/100 km). The survey distances to obtain the recommended minimum of 19 track incidences at different track densities are shown.

Source	Species	Track density (tracks/100 km)	Survey distance for 19 track incidences (km)	Leopard model ^a^		Carnivore on sand intercept model^b^		General carnivore on sand origin model ^c^		Difference (*c* − *b*)∗100∕*c* %
				Density (animals/100 km^2^)	Population estimate	Density (animals/100 km^2^)	Population estimate	Density (animals/100 km^2^)	Population estimate	
Boast & Houser (2012)	Leopard	0.00		0.00	0	−0.13	−13	0.00	0	
Boast & Houser (2012)	Leopard	0.10	19,000	0.05	5	−0.10	−10	0.03	3	410.5
Boast & Houser (2012)	Leopard	0.20	9,500	0.11	11	−0.06	−6	0.06	6	203.5
		0.40	4,750	0.21	21	0.00	0	0.12	12	100.0
Boast & Houser (2012)	Leopard	0.47	4,043	0.25	25	0.02	2	0.14	14	84.6
	Lower extrapolation limit	0.88	2,159	0.46	46	0.15	15	0.27	27	43.5
Boast & Houser (2012)	Cheetah	1.02	1,863			0.20	20	0.31	31	37.1
Boast & Houser (2012)	Cheetah	2.24	848			0.58	58	0.69	69	15.0
Stander (1998)	Leopard	2.62	725	1.38	138	0.70	70	0.80	80	12.3
Bauer et al. (2014)	Lion	3.05	623			0.84	84	0.94	94	10.1
Bauer et al. (2014)	Lion	5.36	354			1.57	157	1.64	164	4.2
Boast & Houser (2012)	Brown hyaena	6.15	309			1.83	183	1.89	189	3.2
Boast & Houser (2012)	Brown hyaena	7.90	241			2.38	238	2.42	242	1.7

A total of 2,273 km need to be surveyed at a track density of 0.88 tracks/100 km to obtain the recommended minimum number of track incidences of 19 (Funston et al., 2010). The required survey distance will increase to 5,000 km at a track density of 0.4 tracks/100 km (Table 7).

## Discussion

Our results show that linear regression through the origin is justified over the more typical linear regression with intercept for the six models we tested. Adding the brown hyaena and leopard data (Table 2) did not alter the slope (*a* = 3.26). The slope of Model 3 General Carnivore on Sand model through origin (*a* = 3.26 ± 0.24; *r*^2^ = 0.98; *t* = 29.169; *P* < 0.001) was very similar to the slope for a small sample of lion and wild dog densities (*a* = 3.28 ± 0.24; *r*^2^ = 0.98; *t* = 13.55; *P* < 0.01) presented by Stander (1998). This further supports the Funston et al. (2010) conclusion that a general model can be used to estimate large carnivore densities from track densities across species and study areas. It is important to use the appropriate model to account for the substrate of the study area, since linear models to estimate large carnivore densities differed significantly on sandy and clay soils (Funston et al., 2010).

Model 3 General Carnivore on Sand through origin provided more conservative leopard population estimates than the Stander (1998) leopard model (Stander, 1998, #75). The Stander (1998) leopard model is from a single study site and based on the assumption that the linear relationship between leopard density and track density holds below the density of 1.45 leopard/100 km^2^ (2.62 tracks/100 km) in his study area. Stander (1998) simulated lower densities by randomly including different numbers of individual leopard in the analysis, but the assumption of a linear relationship between carnivore density and track density may not hold below the sample range.

The valid extrapolation range for Model 3 General Carnivores on Sand through origin exceeds that of Stander (1998), thus allowing carnivore density estimates as low as 0.27 carnivores/100 km^2^ (track density 0.88 tracks/100 km) . Density estimates below this should be considered with caution. Also, this model would not yield negative carnivore density estimates. We therefore conclude that the formula: *observed track density* =* 3.26 ×carnivore density* can be used to estimate densities of large African carnivores using track counts on sandy substrates in areas where carnivore densities are 0.27 carnivores/100 km^2^ or higher. The validity of density estimates below 0.27 carnivores/100 km^2^ (<0.88 tracks/100 km) (Table 7) is questionable, but it may be the best available data to guide conservation. Estimates and trends obtained from track surveys in low density populations should be interpreted with caution.

The potential non-linear relationship with *X* approaching zero (Quinn & Keough, 2002) adds uncertainty to estimates obtained at track densities below 0.88 tracks/100 km. The current models are based on a data set consisting predominantly of lion data points and limited or no data for other large carnivore species. We found a significant linear relationship at low carnivore densities with densities ranging between 0.27 and 0.95 carnivores/100 km^2^. With equal numbers of lion, spotted hyaena, leopard and cheetah, Model 6 Low Density on Sand through origin was not dominated by lion, but all data points were from the Kgalagadi Transfrontier Park. We need data, especially at lower densities, for a variety of large carnivores to improve the current models. The one independent data point for lion density we had, provided a good density estimate. More independent data for different species are required to validate the models.

We recommend that studies using track surveys to estimate carnivore densities provide a data summary with all the relevant data to facilitate recalculation of density estimates. This will ensure that results can be compared among studies that used different models and that density estimates can be recalculated in future if new calibrations become available.

Track surveys are cost effective and can cover large areas. At low carnivore densities there is a trade-off between data quality and survey effort required from track surveys. Selecting an appropriate carnivore survey technique depends on the survey objectives, resources and expertise available, the size of the survey area and expected range of carnivore densities.

## Additional Information and Declarations

### Data Availability

The following information was supplied regarding data availability: The raw data we used in this analysis are included as tables in the article.
